# High-LET-Radiation-Induced Persistent DNA Damage Response Signaling and Gastrointestinal Cancer Development

**DOI:** 10.3390/curroncol30060416

**Published:** 2023-06-07

**Authors:** Kamendra Kumar, Santosh Kumar, Kamal Datta, Albert J. Fornace, Shubhankar Suman

**Affiliations:** 1 Department of Oncology, Lombardi Comprehensive Cancer Center, Georgetown University Medical Center, Washington, DC 20057, USA; kk1264@georgetown.edu (K.K.);; 2 Department of Biochemistry and Molecular & Cellular Biology and Department of Oncology, Georgetown University Medical Center, Washington, DC 20057, USA

**Keywords:** ionizing radiation, heavy-ion radiation, DNA damage response, cancer, senescence, SASP

## Abstract

Ionizing radiation (IR) dose, dose rate, and linear energy transfer (LET) determine cellular DNA damage quality and quantity. High-LET heavy ions are prevalent in the deep space environment and can deposit a much greater fraction of total energy in a shorter distance within a cell, causing extensive DNA damage relative to the same dose of low-LET photon radiation. Based on the DNA damage tolerance of a cell, cellular responses are initiated for recovery, cell death, senescence, or proliferation, which are determined through a concerted action of signaling networks classified as DNA damage response (DDR) signaling. The IR-induced DDR initiates cell cycle arrest to repair damaged DNA. When DNA damage is beyond the cellular repair capacity, the DDR for cell death is initiated. An alternative DDR-associated anti-proliferative pathway is the onset of cellular senescence with persistent cell cycle arrest, which is primarily a defense mechanism against oncogenesis. Ongoing DNA damage accumulation below the cell death threshold but above the senescence threshold, along with persistent SASP signaling after chronic exposure to space radiation, pose an increased risk of tumorigenesis in the proliferative gastrointestinal (GI) epithelium, where a subset of IR-induced senescent cells can acquire a senescence-associated secretory phenotype (SASP) and potentially drive oncogenic signaling in nearby bystander cells. Moreover, DDR alterations could result in both somatic gene mutations as well as activation of the pro-inflammatory, pro-oncogenic SASP signaling known to accelerate adenoma-to-carcinoma progression during radiation-induced GI cancer development. In this review, we describe the complex interplay between persistent DNA damage, DDR, cellular senescence, and SASP-associated pro-inflammatory oncogenic signaling in the context of GI carcinogenesis.

## 1. Introduction

Radiation energy is classified as either ionizing (IR) or non-ionizing radiation. In contrast to non-ionizing radiation, IR radiation has a higher energy and frequency, which enables it to cause ionization upon interaction with biomolecules, resulting in DNA, protein, and lipid damage in the irradiated cells. The extent of IR-induced alterations primarily depends on the absorbed dose and ionization density. The absorbed IR dose is measured in Gray (Gy) units, where 1 Gy represents 1 joule (J) of radiation energy deposited per kilogram (Kg) of matter. Linear energy transfer (LET) is a measure of locally absorbed energy (kiloelectron volts (keV)) per unit length (micrometer (μm)) [1,2]. Exposure to low-LET photons (X-ray or γ rays) results in a homogenous energy deposition throughout the tissue volume, whereas protons (hydrogen nucleus), alpha particles (helium nucleus), and heavy ions (nuclei of atoms with atomic number (Z) > 2) have mass; therefore, they decelerate faster than photons, and the energy deposition rate, or LET, increases as they slow down, leading to the formation of a characteristic Bragg peak before stopping and losing all the energy [2,3]. The energy deposition by particle radiation (proton (H^+^), alpha particle (He^2+^), heavy ions, and neutrons) generally follows a track pattern, where each particle track consists of a central core marked by dense ionization and a penumbra marked by sporadically ionized electrons or δ-rays [4]. Notably, heavy-ion-radiation-induced nuclear interactions in the central core region are also known to produce neutrons, δ rays, and secondary charged particles of varying LET [5]. The physical characteristics of different IR types are summarized in Table 1. Solar particle events (SPEs) and galactic cosmic radiation (GCR) are the two primary sources of IR in deep space, where protons make up about 90% and 87%, respectively, of the SPE and the GCR [6,7]. In addition to protons, alpha particles (8–10%) and heavy ions (1–2%) are also present in the deep space radiation environment [7]. Despite being a minor constituent of space radiation, heavy-ion radiation is considered a greater threat to astronauts’ health due to its ability to penetrate through spacecraft shielding and its densely ionizing characteristics [8].

**Figure 1:**
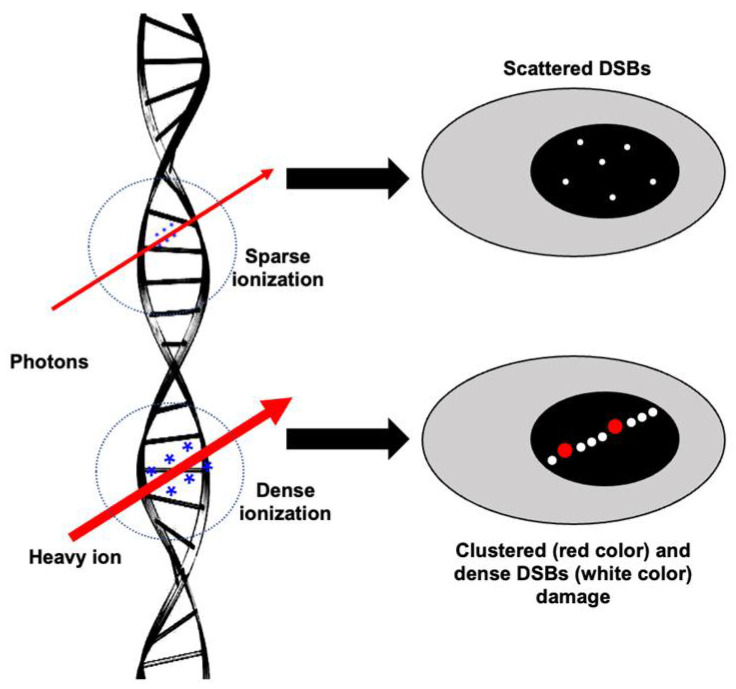
Illustration of ionization events and subsequent damage to DNA after low-LET (photon) vs. high-LET (heavy-ion) radiation. The size of * (in blue) represents the ionization density after photon and heavy ion irradiation. Scattered DSBs are marked with small white dots. Dense DSBs are marked with big white dots, and a red dot represents complex damage (SSB, DSB, and oxidative DNA damage in close proximity).

**Figure 2:**
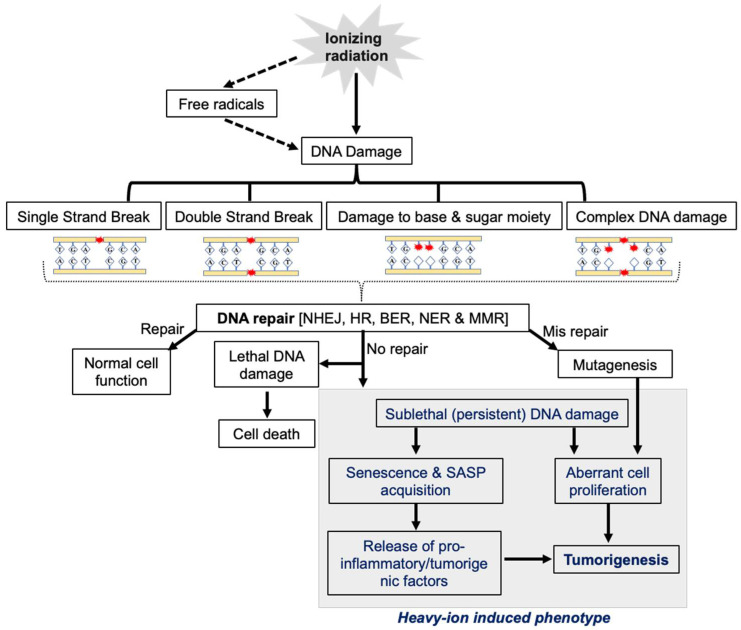
The central role of DDR in heavy-ion-radiation-induced persistent DNA damage, senescence/SASP, and GI carcinogenesis.

Both low- and high-LET radiation can act directly or indirectly on its biological targets, including DNA. The ion pairs and free radicals are produced in the DNA by its direct effect, whereas water or other molecules surrounding DNA are ionized and form free radicals, acting as intermediaries causing DNA damage, and refer to an indirect effect. Therefore, both the direct and indirect actions of radiation involve the formation of free radicals [9]. IR-induced DNA damage includes base damage, single-strand breaks (SSBs), and double-strand breaks (DSBs) [10,11,12] (Figure 1). Based on the presence of these damage types in close proximity, they may also form clustered DSBs (>2 DSBs) and complex DNA damage (DSBs with SSBs and base/nucleotide damage) [13]. Qualitative and quantitative differences in the damage after photon and heavy-ion radiation have been noted, where the same dose of photon exposure results in fewer DSBs compared to high-LET heavy ions, which lead to clustered damage with more DSBs [14,15,16]. High-LET-radiation-induced clustering of radicals is believed to be the fundamental reason for the formation of locally multiply damaged sites (LMDSs) [17]. Complex DSBs defined as clusters of base damages and SSBs near the DSB sites are more difficult to repair than infrequent DSBs and SSBs, and the complexity of DNA damage clusters generally rises with LET [18,19]. Furthermore, the misincorporation of nucleotides during the DNA repair process could also lead to some additional DNA damage in IR-exposed cells [20]. After DNA damage, the DDR is triggered by the recruitment of damage sensor proteins, followed by the accumulation of DNA repair proteins at the damage sites [21]. DDR signaling is also equipped with cell cycle checkpoint control that can arrest damaged cells at various points in the cell cycle, allowing DNA repair enzymes to repair the damaged DNA before its progression through replication and cell division [22]. Finally, the DDR also determines whether the repair process has been completed or not [23]. Cells with unrepaired or misrepaired DNA either undergo cell death, senescence, or proliferation that could culminate in tissue toxicity, an accelerated aging phenotype marked by cellular senescence, and/or carcinogenesis [24,25] (Figure 2).

## 2. Role of DNA Repair Machinery in Cellular Response to IR

**Figure 3:**
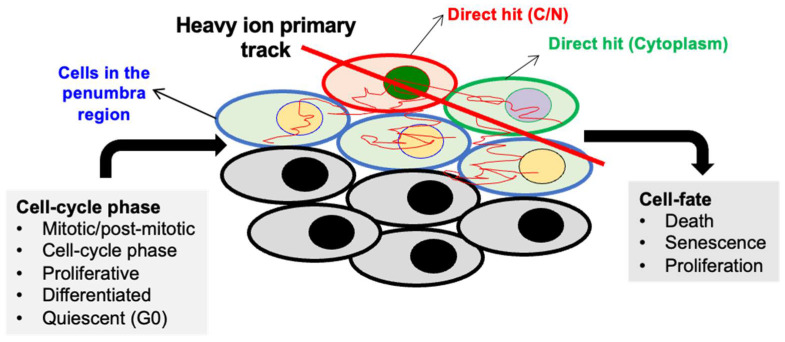
Variability in radiation response (cell fate) due to cell cycle phase at the time of irradiation and heterogeneous damage caused by heavy ions in the cells located in the core and penumbra regions The heavy-ion track is divided into “core” and “penumbra” regions based on the deposited energy density. The penumbra is the area near the edge of an ion beam track where the deposited energy density changes according to the distance from the primary ion track.

Cellular responses and fates after IR exposure vary greatly, which are believed to depend on the cell cycle stage [26], radiation dose [27], LET [28], and damage quality, i.e., DSB, clustered DSB, and complex damage [13,29] (Figure 3). The DDR is a multifaceted signaling network that involves DNA damage sensing, the recruitment of DNA repair proteins, the activation of cell-cycle checkpoints, and the preservation of genomic integrity via the induction of cell death [30]. DNA repair pathways are the core component of the DDR [31], and its optimal functioning is crucial after IR-induced DNA damage to maintain genomic integrity [32]. Five major DNA repair pathways are known to exist in mammalian cells, i.e., (i) non-homologous end joining (NHEJ); (ii) homologous recombination (HR); (iii) nucleotide excision repair (NER); (iv) base excision repair (BER); and (v) mismatch repair (MMR) [33]. Specifically, in cases of IR-induced DNA damage, the DSB is considered the most difficult damage to repair, and the NHEJ and HR pathways are two primary modes of DSB repair [34,35]. HR-pathway-associated proteins include the MRN complex (Meiotic recombination 11 (MRE11), RAD50, and Nijmegen breakage syndrome protein 1 (NBS1)), breast cancer susceptibility proteins (BRCA1 and 2), ATM (Ataxia telangiectasia mutated), and ATR (Ataxia telangiectasia and Rad3-related) [36,37]. The NHEJ repair is mediated by the DNA-PKcs (DNA-dependent protein kinase catalytic subunit), XRCC5 (X-ray repair cross-complementing protein 5, commonly known as Ku86), XRCC6 (X-ray repair cross-complementing protein 6, commonly known as Ku70), XRCC4 (X-ray repair cross-complementing 4 (XRCC4), and ligase IV [38]. In addition, an alternate NHEJ (alt-NHEJ) pathway also exists in some cell types, which is initiated by PARP1 (Poly (ADP-Ribose) Polymerase 1), together with DNA ligase [39]. Because NHEJ requires the modification of incompatible DNA ends prior to ligation, it is regarded as a potentially error-prone repair pathway [40].

In addition to potentially lethal DSB formation, IR exposure also results in reactive oxygen species (ROS)-mediated base and nucleotide damage in the DNA [11,41]. Oxidatively modified DNA bases and sugar moieties are repaired through the BER and NER pathways [42,43]. For example, the most common oxidative DNA damage, i.e., 8-Oxo-2′-deoxyguanosine (8-Oxo-dG), is repaired by BER, where 8-Oxo-dG is first recognized and excised by 8-Oxo-dG DNA glycosylase (OGG1), resulting in the formation of an abasic (AP) site. Further, excision of the AP site by AP endonuclease 1 (APE1) results in the formation of SSB, which is repaired by PARP1 and DNA ligases [44]. NER is associated with repairing other forms of DNA damage, such as pyrimidine dimers commonly formed after ultraviolet (UV) exposure [45], whereas MMR plays an important role in the rectification of polymerase misincorporation errors [46].

The choice of a DNA repair pathway depends on the location and complexity of DNA damage [47]. The DSBs in the part of chromatin with active replication and transcription, i.e., euchromatin, are handled mainly by both the NHEJ and HR pathways, whereas in the condensed part of chromatin, i.e., heterochromatin, HR-mediated repair is less feasible [48]. Significantly slower DSB repair in heterochromatin is attributed to its structural complexity [49]. In actively proliferating cells, such as GI-epithelial cells, the choice of DSB repair pathway is likely to depend on the complexity of DNA damage [11,50,51]. The complex DSBs generated after heavy-ion irradiation are difficult to repair by NHEJ [52,53]. Moreover, the short DNA fragments formed near heavy-ion-induced complex DSBs have been demonstrated to inhibit NHEJ activity, mainly due to the difficulty in recruiting Ku70/86 to the damage site [54,55]. The 53BP1 protein plays a significant role in determining the DSB repair pathway choices by promoting chromatin compaction and inhibiting DSB-end resection by blocking DNA nuclease access to the DSB site [56]. Chromatin environments that block DNA-end resection are considered suitable for Ku70/86 retention at DNA ends, which could promote NHEJ-mediated repair. Therefore, 53BP1 foci formation is seen at DSBs with an ongoing DDR [50]. Conversely, factors such as RAD51 and BRCA2 are known to antagonize 53BP1 and lead to the activation of the HR pathway [57]. Using NHEJ and HR-deficient cells, the greater relevance of the HR pathway in heavy-ion-induced DNA repair has been demonstrated [53].

The GI epithelium consists of both quiescent, actively dividing, and differentiated cells [58]. DNA repair pathway choice and functioning also vary greatly during different phases of the cell cycle [59,60]. A recent study on DNA damage checkpoints has shown that in early G1 and G2, checkpoints are stringent and the arrest duration is proportional to the extent of DNA damage; however, checkpoint stringency is somewhat relaxed during the S phase [60]. This suggests that checkpoints are phase-dependent, and the exact cell-cycle position at the time of radiation could determine if cells are allowed to progress through the cell cycle even without completing the DNA repair process. HR repair is considered less error-prone (less mutagenic) than NHEJ but is only functional during the S/G2 phase, whereas NHEJ is active throughout the cell cycle [61]. Hence, despite its error-prone nature, NHEJ is responsible for the majority of DSB repair after IR exposure [35]. Moreover, MMR pathway activation in the S-phase of the cell cycle is also known to suppress HR when excessive mismatched nucleotides are present [62].

DNA damage leading to mutagenesis and the subsequent activation of oncogenes and/or inactivation of tumor suppressor genes have established roles in cancer progression. An increased IR-induced risk of carcinogenesis often displays increased mutagenesis in genomic DNA [63]. However, heavy-ion radiation at low fluence can harm cells in multiple distinct ways, as depicted in Figure 3, i.e., (i) a direct hit to nuclear DNA; (ii) a direct hit to the cytoplasm or cytoplasmic organelles of a cell; (iii) an indirect (secondary) hit to the nearby cells; and (iv) the emission of signals from the neighbor (bystander) of a directly or indirectly hit cell. The current understanding of cellular responses after heavy-ion exposure suggests that a direct nuclear traversal by a heavy-ion track would result in cell death [51,64]. However, sublethal damage caused by secondary δ-rays is attributed to delayed tissue effects, including increased cancer risk [65,66]. Because an in vivo tissue is a 3D assembly of cells, signal emission from a directly hit cell’s neighbor would represent an amplifier effect; thus, a greater role for non-targeted effects (NTEs) is expected at low-dose, low-fluence exposure in tissues compared to in vitro 2D cultures [67,68]. Depending on the outcome of DNA repair, cells either survive normally or, if DNA damage is beyond repair capacity, cell death is initiated. However, if a cell with sub-lethal DNA damage survives and replicates, the likelihood of genomic instability, cellular transformation, and carcinogenesis could increase. Additionally, a stringent DDR can also promote a permanent growth arrest via activation of the cell cycle checkpoint, causing increased accumulation of senescent cells [69,70]. Recent studies analyzing the late effects of low-LET and heavy-ion exposure have shown an increased number of both senescent and proliferative (mitotic) cells in the mouse GI epithelium, where cell differentiation, migration, and autophagy were significantly reduced [50,71,72,73,74,75,76,77]. Moreover, a subset of heavy-ion-induced senescent cells acquired a senescence-associated secretory phenotype (SASP) that could potentially drive pro-inflammatory and oncogenic signaling in nearby proliferative cells [71,72].

## 3. DDR Alterations in GI Cancer

The DDR ensures the transmission of undamaged DNA to the daughter cell during cell division, and any malfunction in this critical pathway would contribute to the loss of genomic integrity. DDR alterations are one of the known hallmarks of CRC development, often detected in the form of point mutations and/or copy-number alterations such as loss of heterozygosity (LOH) in somatic cells [78,79,80]. Integrative genomic analysis at various stages of CRC development has shown increased mutagenesis during benign to invasive cancer progression. In cases of early-onset CRC patients, the mutation rates were 4.0% and 12.2% in the polyps and cancer samples, respectively [81]. Moreover, the frequency of somatic DDR gene alterations in CRC has been reported earlier [82,83]. Up to 15–20% of sporadic human colorectal cancers carry alterations in the DDR genes [84]. CRC tumors have been reported to harbor a higher global mutation rate that also includes DDR pathway genes, where MMR pathway alterations are very frequent [82]. A recent study also reported frequent mutations in DNA repair genes (MLH1, BRCA1, BRCA2, CHEK2, BLM, and NTHL1) in sporadic CRC [81]. In addition to the core DDR genes, 43% of CRCs also display mutations in the TP53 (tumor suppressor p53) gene, which is a key regulator of the IR-induced DDR [85]. Moreover, reduced tumor suppression by p53 is also reported in CRC as a consequence of changes in its upstream signaling partners, i.e., ATM and DNA-PKcs. Similar to human CRC, mouse models of CRC also display DDR alterations [86]. For example, Msh2-deficient colonic epithelial cell hyper-proliferation has been observed in *Msh2*^−/−^; *Apc*^min/+^ mice [87]. In addition, epigenetic alterations, including the silencing of DDR genes due to promoter hypermethylation, have also been reported during CRC pathogenesis [88]. Hypermethylation-mediated silencing of MLH1 is common in human CRC [89]. Moreover, altered DNA methylation in approximately 40% of the p53 pathway gene promoters has also been reported, along with the frequent down-regulation of p16 and p21 in human CRC [89]. Similar epigenetic alterations in mouse and human colorectal cancer are also observed [90,91,92]. Therefore, both genetic and epigenetic alterations in the DDR are important in GI cancer development.

## 4. DDR Alterations in Heavy-Ion-Radiation-Induced GI-Carcinogenesis

The adverse effects of IR are more pronounced in rapidly renewing tissues, such as the GI epithelium. The GI mucosa is composed of tightly regulated epithelial cells with a high turnover rate. A well-coordinated cellular homeostasis is required to maintain normal cell proliferation, differentiation, and cell death along the crypt-villi axis to maintain the integrity of the GI mucosa [58]. Mouse GI epithelial cells are replaced every few days along the crypt-villus axis, where stem cells reside at the bottom of the crypt and are considered the cells of origin for CRC development [93,94]. Therefore, IR-induced damage to the stem cell compartment at the crypt base could likely affect cell replacement dynamics due to non-replacement, slow replacement, or replacement with transformed cells.

Colorectal cancer (CRC), a type of gastrointestinal (GI) cancer, is the third most common cancer in the U.S. (https://seer.cancer.gov/statfacts/html/colorect.html, accessed on 20 February 2023). In epidemiological studies, such as those of Japanese A-bomb survivors, occupational radiation workers, and radiotherapy patients, the relationship between excess GI cancer incidence and IR exposures is well documented [95,96,97,98]. GI cancer was reported as the third most frequent solid cancer in A-bomb survivors [98]. Considering the high frequency of CRC in the general U.S. population, the space-radiation-induced increase in cancer incidence among astronauts during long-term deep space missions, such as to Mars, is projected to exceed the National Aeronautics and Space Administration’s (NASA) current limit of 3% REID (risk of exposure-induced death) from cancer [99]. Current projections for GI cancer risk to astronauts during and after a Mars mission are being developed using in vitro and in vivo models exposed to simulated space radiation [99,100,101]. To understand the DDR changes linked to potentially higher GI tumorigenic risk after heavy-ion exposure, a series of studies at the cellular, genomic, transcriptomic, proteomic, and metabolomic levels have been conducted [11,50,71,72,73,74,75,76,77,102,103,104,105,106,107,108,109,110] and are summarized below:

### 4.1. In Vitro Studies

Exposure to heavy-ion radiation has been shown to cause neoplastic transformation and promote cell proliferation in many cell culture models [111,112,113]. A non-transformed two-dimensional (2D) human colon epithelial cell (HCEC) model was used by Roig et al. to demonstrate the tumorigenic effects of heavy-ion (^56^Fe) radiation [108]. Months after heavy-ion irradiation, a wide variety of karyotypes were observed in transformed HCECs, where approximately 40% of transformed HCECs showed partial loss of chromosomes 13p and 17p. The loss in chromosome 17p was also associated with the downregulation of the tumor suppressor p53 pathway [109], which is known to inhibit the genomic and phenotypic changes associated with carcinogenesis through regulation of the DDR [109]. Altogether, in vitro studies using HCECs indicate a higher risk of CRC development involving DDR alterations after heavy-ion irradiation.

### 4.2. Animal Model Studies

Dose and radiation quality are considered the key determinants of IR-induced late GI alterations, including tumor development [73,104,107]. Somatic mutations in the Adenomatous polyposis coli (*Apc*) gene are considered one of the key precursor events for the development of sporadic CRC in humans and are seen in most pre-neoplastic adenomatous polyps [114]. Therefore, *Apc*-mutation-based murine models of GI-cancer such as *Apc*^Min/+^, *Apc*^1638N/+^, and CDX2P*Apc*^flox/+^ mice have been frequently employed to assess the GI-cancer risk after simulated space radiation exposures [73,103,104,105,106,110]. Multiple studies using photons, protons, heavy ions (C, O, Si, and Fe), and sequentially delivered H, He, O, and Si beams have been conducted to investigate the dose–response, radiation quality factor, and effect of dose rate, sex, and age on GI cancer risk [104,105,107]. The carcinogenic potential of heavy ions has been unequivocally observed with increased GI tumorigenesis and carcinoma progression relative to γ-rays. RBE values ranging from 3.7 to 8 for GI tumorigenesis and 8 to 42 for carcinoma progression have previously been reported [104,115,116]. Heavy-ion IR-induced GI tumorigenesis is primarily dependent on LET (peak tumorigenesis at 70 keV/micron) and dose but is generally dose-rate-independent [104,105]. A recent study comparing individual heavy-ion radiation vs. simulated galactic cosmic radiation (GCRsim)-induced GI tumorigenesis also revealed the predominant role of heavy-ion radiation in GCR-induced GI carcinogenesis [107]. The acquisition of spontaneous or radiation-induced mutations in the functional *Apc* allele is generally considered an important early event for GI tumorigenesis in *Apc* mouse models [117,118,119]. In addition to the expected loss of heterozygosity (LOH) in *Apc*, a persistent decrease in expression of tumor suppressor p53 and increased somatic mutations in the p53 gene have been noted in heavy-ion-induced colon tumors in mice [110]. Moreover, when an antagonist of p53 signaling and a known oncogene, i.e., Wip1 (wild-type p53-induced phosphatase 1), was knocked out in *Apc*^min/+^ mice, IR-induced GI tumorigenesis was abrogated [102]. This also suggested a critical role for DDR and p53 signaling in heavy-ion IR-induced GI tumorigenesis. While gene mutations are often required for tumor initiation, the “two-hit model” of carcinogenesis suggests the role of both intrinsic (genetic) and extrinsic (tissue microenvironment/systemic/epigenetic) signaling events in adenoma to carcinoma progression [76,120,121,122]. In cases of heavy-ion exposure, bystander cells adjacent to the directly hit cells are more likely to survive with sublethal DNA damage, which is believed to play a part in the cancer development process [123]. Assessments of late-progressive signaling alterations and associated changes in cellular phenotypes in the GI epithelium have revealed ongoing chronic oxidative stress [50,72,77], reductions in DNA repair capacity [11,50], persistent DNA damage [11,50], increased mutagenesis [110], increased expression of the senescence-inflammatory response [71,72,110], increased accumulations of inflammatory mediators [72], and activation of oncogenic (β-catenin, mTOR, and PI3K-Akt) signaling [71,73,76]. These findings suggest a key role for ongoing DDR dysfunction, resulting in increased mutagenesis and accumulations of senescence and SASP cells that subsequently drive pro-inflammatory and pro-oncogenic signaling. This hypothesis is also supported by the higher number of GI-carcinoma cases in space-radiation-exposed mice [107,115].

## 5. Persistent DDR, Cellular Senescence, and Accumulation of SASP Cells

Higher DNA damage, an altered DDR, and reduced DNA repair capacity observed after heavy-ion irradiation compared to photon radiation are attributed to its higher RBE (>1) for cell death, mutagenesis, accelerated aging, and cancer risk [11,107,116,124]. Sub-lethally damaged bystander cells are likely to display chronic oxidative stress alongside ongoing or persistent DNA damage and a higher risk of mutagenesis and cancer development. Exposure to both low-LET and heavy-ion radiation has been associated with increased ROS production, oxidative stress, and DNA damage [125,126]. Human skin fibroblast exposed to C-ions displayed a secondary wave of oxidative damage two weeks post exposure, which indicates the role of intrinsic cellular mechanisms in the onset and persistence of late oxidative stress [127]. Reports demonstrating the propagation of oxidative stress from progenitor cells to progenies suggest that initial non-lethal damage in bystander GI epithelial cells could persist and amplify with ongoing oxidative stress [128,129,130,131,132]. Multiple mouse model studies from our group have demonstrated that exposure to heavy-ion (C, Si, or Fe) radiation causes significantly higher persistent oxidative stress (2 to 12 months) in the mouse GI epithelium relative to γ-rays [50,72,77]. The persistent increase in ROS production after heavy-ion exposure was attributed to mitochondrial dysregulation and increased NADPH oxidase activity. Additionally, decreased antioxidant enzyme activities in the GI tissues of heavy-ion-irradiated mice were also noted [50]. Higher chronic oxidative stress and increased levels of 8-Oxo-dG (a marker of oxidative DNA damage) after heavy-ion exposure relative to low-LET radiation have also been reported in GI tissues [11,78]. Interestingly, in addition to indirect-effect-mediated 8-Oxo-dG formation, the direct action of high-LET radiation has also been reported to cause 8-Oxo-dG accumulations [9]. Importantly, a higher accumulation of 8-Oxo-dG has also been reported in CRC [133].

**Figure 4:**
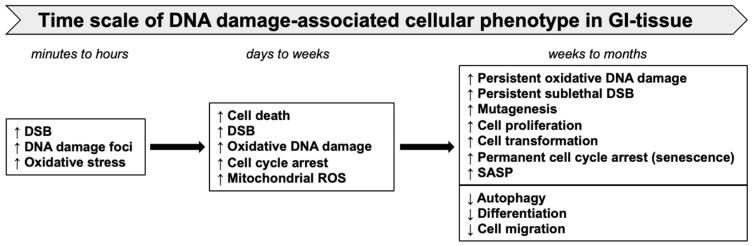
Time scale of heavy-ion-induced oxidative stress, DNA damage, and cellular phenotypes in GI tissue.

The timescale of DNA-damage-associated cellular changes and consequent phenotypes observed in GI tissue after sublethal heavy-ion radiation exposure is depicted in Figure 4. The DSB repair kinetics of cells irradiated with heavy ions are usually slower than those of low-LET radiation [134]. The size of the γH2AX and 53BP1 foci associated with DNA damage caused by high-LET heavy ions is reportedly larger than that of γ-rays and often denotes a complex DSB [135]. The 53BP1 foci indicate an ongoing DDR with NHEJ as a primary DNA repair pathway in the case of heavy-ion-induced persistent DSBs [50]. Persistent DNA damage after heavy ions was also reported using a 3D colon organotypic culture [108]. Additionally, a progressive persistence (2 to 12 months post exposure) of DSB and oxidative base damage in the GI-epithelial cells of heavy-ion-exposed mice had been reported earlier [11,71]. Significantly higher accumulations of 8-oxodG in heavy-ion-irradiated mouse intestinal samples indicated a higher oxidative DNA damage compared to the same dose of γ rays [11,77]. Increased complex DNA damage marked by co-localized γ-H2AX/phospho-DNA-PKcs was observed after heavy-ion exposure in two-dimensional non-transformed human colon epithelial cells [108]. Furthermore, the increased presence of γH2AX and 53BP1 foci long-term after heavy-ion exposure indicated persistent DSB and ongoing DDR.

Intrinsic alterations in the DDR and ongoing DNA damage caused by persistent oxidative stress could potentially amplify the number of DNA damage sites with time. Activation of the DDR-mediated cell-cycle checkpoint in GI-stem cells with sublethal DNA damage might result in permanent growth arrest and cellular senescence. Persistent DSBs and DDRs observed long-term after heavy-ion exposure were associated with no significant change in the number of dying cells (marked by the TUNEL assay), an increased GI-epithelial cell proliferation (marked by phospho-histone H3, Ki67, and PCNA), [50,71,76,77], and an increase in the accumulation of senescent cells (marked by SA-β-gal, p16, and Glb1) [71,72]. Stress-induced senescence (SIS) is generally independent of telomere erosion and classified as either oncogene-induced senescence (OIS) or DDR-induced senescence. Indications for both OIS- and DDR-induced senescence in the promotion of heavy-ion-induced GI cancer have been observed [71,72,110]. However, the initiation of SIS appears to be DDR-induced in the beginning, and later on, the activation of OIS is likely to follow. Additionally, cells displaying persistent DNA damage signaling are more likely to secrete inflammatory cytokines [136] and display the SASP [71,72]. DDR proteins appear to be required for the initiation and persistence of inflammatory cytokine secretion from irradiated cells; for example, ATM (a key DDR component) is an essential factor for IL-6 secretion in senescent cells [136]. Studies using heavy-ion-irradiated Lgr5-EGFP-IRES-creERT2 mice expressing reporter EGFP and CreERT2 fusion proteins in Lgr5+ intestinal stem cells demonstrated the acquisition of senescence and SASP as a result of persistent DNA damage [71]. Heavy-ion-induced SASP in mouse GI tissue was also accompanied by an increased secretion of inflammatory cytokines, including IL-6 [71]. Additionally, both DDR activity and IL-6 are often elevated in CRC. Therefore, apart from regulating DNA repair and cell-cycle progression, DDR signaling in cells with persistent DNA damage could influence the tissue microenvironment via provoking robust SASP signaling [136].

## 6. SASP Signaling in GI Cancer Progression

**Figure 5:**
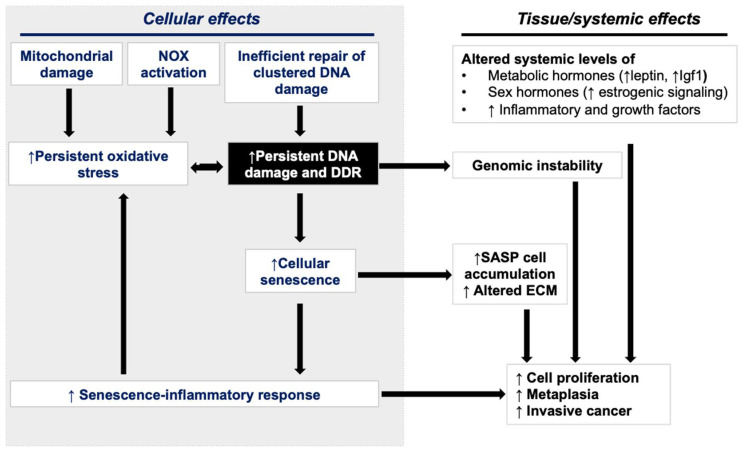
Interplay between cellular and exogenous systemic factors in the promotion of GI carcinogenesis after heavy-ion exposure.

Increased proliferation of mutation-bearing cells is one of the early precursor events in the multistage model of carcinogenesis [137]. Mechanistically, GI carcinogenesis observed in response to heavy-ion exposure is likely fueled by both cellular and systemic effects (Figure 5). Carcinogenic events observed in GI epithelial cells include persistent DNA damage and a consequent increase in both cell proliferation and senescence. The accumulation of SASP in a subset of senescent GI cells has the potential to drive pro-inflammatory and pro-oncogenic signaling pathways [71,72,110]. CRC development occurs via aberrant crypt foci (ACFs) to adenoma-to-carcinoma progression, and heavy-ion exposure is known to accelerate carcinoma progression [138], whereas SASP signaling is also known to accelerate GI-cancer development [107,115]. Further, heavy-ion-radiation-induced late GI epithelial alterations could also be influenced by systemic factors such as (i) altered serum levels of metabolic and sex hormones; (ii) systemic pro-inflammatory factors; and (iii) pro-growth signaling [71,76,104,110,139]. The somatic gene mutation is considered a rate-limiting step, but myriad other intrinsic and extrinsic factors could drive cancer development [120]. DDR alterations could result in both somatic gene mutations and the activation of pro-inflammatory and oncogenic SASP signaling implicated in carcinogenesis. SASP-mediated microenvironmental changes and systemic inflammatory and pro-growth effectors are known to accelerate adenoma to carcinoma progression during CRC development [140].

## 7. Space-Radiation-Induced GI-Cancer Risk Reduction through DDR Modification

**Figure 6:**
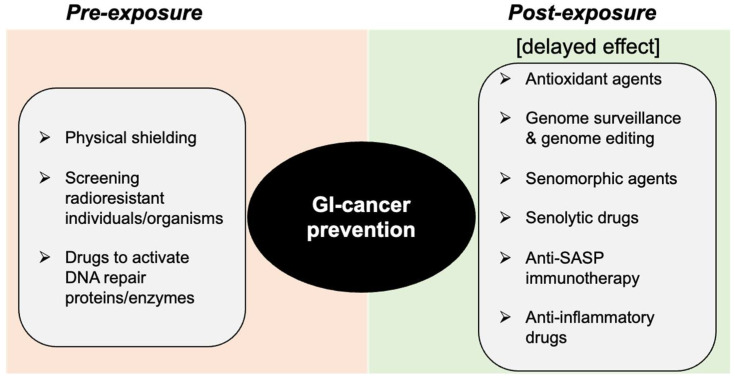
Schematic depiction of the DDR modulation approach for prevention of heavy-ion space-radiation-induced GI carcinogenesis.

Persistent genotoxic and oncogenic stress following heavy-ion exposure could trigger cellular senescence/SASP that could accelerate carcinoma progression in the GI tract. Since the DDR is key for the acquisition of SASP, interventions pre- (protection), during, or post exposure (mitigation) are possible to modify the DDR and reduce GI-cancer risk (summarized in Figure 6). Reducing the total IR dose from high-LET radiation in space is expected to reduce potentially carcinogenic DNA damage induction, and low-level DNA damage is possibly more repairable by DNA repair machinery following the DDR. Therefore, efficient heavy-ion dose reduction by the use of radiation shielding would be best to reduce GI-cancer risk among astronauts, while dose reduction below the recommended safe limits has yet to be achieved [141].

Eukaryotic model organisms resistant to IR-induced damage often display efficient and robust DNA damage repair machinery [142,143,144]. Additionally, genome-wide RNA interference (RNAi)-based screening in *Caenorhabditis elegans* also suggested the role of DSB repair genes and proteins in determining radiosensitivity in multicellular organisms [145]. No data on human sensitivity to whole-body heavy-ion exposure are available; however, IR-induced normal GI-tissue toxicity after radiotherapy varies greatly among cancer patients [146], which is often attributed to variations in dose delivery, genotype, age, and sex. However, a recent report suggested an association between high-baseline DNA damage and severe secondary effects of IR exposure [147]. Therefore, approaches to screen individuals with low-baseline DNA damage and efficient DDR machinery would potentially reduce GI cancer risk after space radiation exposure. Further investigations are warranted to understand the molecular determinants of individual radiosensitivity after heavy-ion exposure. Meanwhile, therapeutics with known modulatory effects on the DDR could also effectively trigger cell death in mutation-bearing cells [148,149] and therefore reduce the late risk of cancer development. For example, molecules such as curcumin and resveratrol have been implicated in the modulation of the DDR in healthy cells. The curcumin-mediated prevention of DNA damage involves improvements in DSB repair capacity along with increased expression of proteins involved in NHEJ and BER [150]. Similarly, resveratrol has been reported to enhance the non-mutagenic repair of DNA damage in irradiated stem cells [151].

IR-induced persistent oxidative DNA damage could trigger a DDR, which can be alleviated by antioxidants [152]. A significant heavy-ion-induced decrease in plasma antioxidant status has also been reported [153]. A decline in antioxidant status is a common phenomenon during accelerated aging and is often associated with an increased risk of cancer, including CRC, and emerging data on many dietary antioxidants suggest their cancer-preventive efficacy. Antioxidants have been shown to reverse the accumulation of damaged DNA [154]. Therefore, heavy-ion-induced compromises in the antioxidant system can be prevented by antioxidant supplementation. An antioxidant and anti-inflammatory drug, CDDO, has shown promising efficacy against space-radiation-induced G carcinogenesis in mice [110].

Moreover, DSB monitoring for health resilience in space has been suggested recently [148]. Genomic surveillance and editing approaches are another potential alternative to manipulating the DDR outcome in a post-exposure scenario, where mutations could be detected and reversed to the normal genotype. However, older genome editing strategies, such as the CRISPR–Cas9 approach, is based on a DSB-mediated gene editing, which is often associated with undesired indels, gene rearrangements, activation of the DDR, and p53 signaling [155]. In contrast, in the DSB-mediated genome editing approach, newly developed base editor (BE) and prime editor (PE) systems are designed to precisely edit genomes without creating a DSB. Recently, the prime editor system has demonstrated the ability to edit any mutation with desired changes up to dozens of base pairs [156,157]. While this emerging technology is far from human applications, it is expected to evolve in its in vivo feasibility, which might have future implications for the development of a potential mitigation approach against space-radiation-induced GI carcinogenesis.

In addition to the use of anti-senescence strategies, the use of anti-inflammatory drugs to mitigate heavy-ion-induced cancer risk is also suggested. However, an ideal anti-inflammatory agent with good efficacy has yet to be developed. Aspirin (a well-known anti-inflammatory drug) displayed no effective prevention from space-radiation-induced GI carcinogenesis, while a noticeable decrease in serum prostaglandin-E2 (PGE2) was noted [106]. Aspirin primarily exerts its action through the reduction in PGE2, while heavy-ion exposure causes accumulations of many other pro-inflammatory and pro-carcinogenic factors, including IGF1, leptin, IL-6, and IL-8, in GI tissue [71,72,76]. Therefore, an agent with an anti-inflammatory effect exerted through multiple pathways is deemed more suitable for future countermeasure studies.

Heavy-ion-induced DDR checkpoint activation results in the accumulation of senescent cells in the GI tissues [71,72]. Due to their intrinsic resistance against apoptosis, these senescent cells are likely to continue accumulating in the presence of persistent oxidative stress, and a subset of these cells could acquire SASP. Secretion from SASP cells can promote secondary senescence in nearby cells and could result in pro-inflammatory/carcinogenic changes in the GI tissue microenvironment. Recently, a “serotherapeutic approach” to reduce space-radiation-induced GI-cancer risk has been proposed [158], which includes: (a) inhibition of secondary senescence using senomorphic (or senostatic) drugs; (b) cell death induction in SASP cells using senolytic or immunotherapy strategies; and (c) using SASP neutralizing antibody (SNmAb) to mitigate the pro-inflammatory/carcinogenic effects of SASP factors. Metformin, a commonly used anti-diabetic drug with senomorphic properties, has been recently reported to protect against heavy-ion-induced GI cancer [159]. Future studies using the post-exposure administration of senomorphic, senolytic, and SNmAb are required to establish the GI-cancer mitigation ability of senotherapeutics against space-radiation-associated GI cancer risk.

## 8. Conclusions

The coexistence of accelerated senescence/SASP and the proliferation of a mutation-bearing cell potentially depend on heavy-ion-induced persistent DNA damage and DDR malfunction. Therefore, it is plausible that DDR regulation leading to an improved mutation-free DNA repair and decreased accumulation of stress-induced senescent/SASP cells will decrease the risk of GI cancer after space radiation exposure. Several DDR modulation strategies for pre- and post-exposure settings have been proposed that need to be evaluated using in vitro and in vivo studies. Research along these lines has the potential to deliver suitable strategies to protect astronauts from space-radiation-induced GI and other carcinogenic as well as non-carcinogenic adverse health effects.

## Figures and Tables

**Table 1:** Basic differences in the physical characteristics of different IR-types.

IR-Types	Physical Characteristics			
	Energy	Mass	LET (keV/μm)	Charge
Photon (X-or γ-rays)	Yes	No	Low	X-ray (Negative); γ-rays (Neutral)
Proton (H^+^, i.e., nucleus of H atom)	Yes	Yes (Equivalent to proton)	Low to intermediate	Positive
Alpha particle (He^2+^)	Yes	Yes (Equivalent to helium nucleus)	Intermediate to high	Positive
Heavy-ion (Z > 2)	Yes	Yes (Equivalent to nucleus of an atom)	Intermediate to high	Positive
Neutron	Yes	Yes (Equivalent to neutron)	Intermediate to high	Neutral

## Data Availability

Not applicable.
